# Cellular heterogeneity and repolarisation across the atria: an in silico study

**DOI:** 10.1007/s11517-022-02640-x

**Published:** 2022-09-15

**Authors:** Jordan Elliott, Luca Mainardi, Jose Felix Rodriguez Matas

**Affiliations:** 1 Department of Chemical and Material Engineering, Politecnico Di Milano, 20133 Milan, Italy; 2 Department of Electronic, Information and Bioengineering, Politecnico Di Milano, 20133 Milan, Italy

**Keywords:** Atrial fibrillation, Heterogeneity, Electrophysiology, Repolarisation, Electrotonic coupling

## Abstract

Mechanisms of atrial fibrillation and the susceptibility to reentries can be impacted by the repolarization across the atria. Studies into atrial fibrillation ignore cell-to-cell heterogeneity due to electrotonic coupling. Recent studies show that cellular variability may have a larger impact on electrophysiological behaviour than assumed. This paper aims to determine the impact of cellular heterogeneity on the repolarization phase across the AF remodelled atria. Using a population of models approach, 10 anatomically identical atrial models were created to include cellular heterogeneity. Atrial models were compared with an equivalent homogenous model. Activation, APD90, and repolarization maps were used to compare models. The impact of electrotonic coupling in the tissue was determined through a comparison of RMP, APD20, APD50, APD90, and triangulation between regional atrial tissue and the single cell populations. After calibration, cellular heterogeneity does not impact atrial depolarization. Repolarization patterns were significantly impacted by cellular heterogeneity, with the APD90 across the LA increasing due to heterogeneity and the reverse occurring in the RA. Electrotonic coupling caused a reduction in variability across all biomarkers but did not fully remove variability. Electrotonic coupling resulted in an increase in APD20 and APD50, and reduced triangulation compared to isolated cell populations. Heterogeneity also caused a reduction in triangulation compared with regionally homogeneous atria.

**Graphical abstract:**

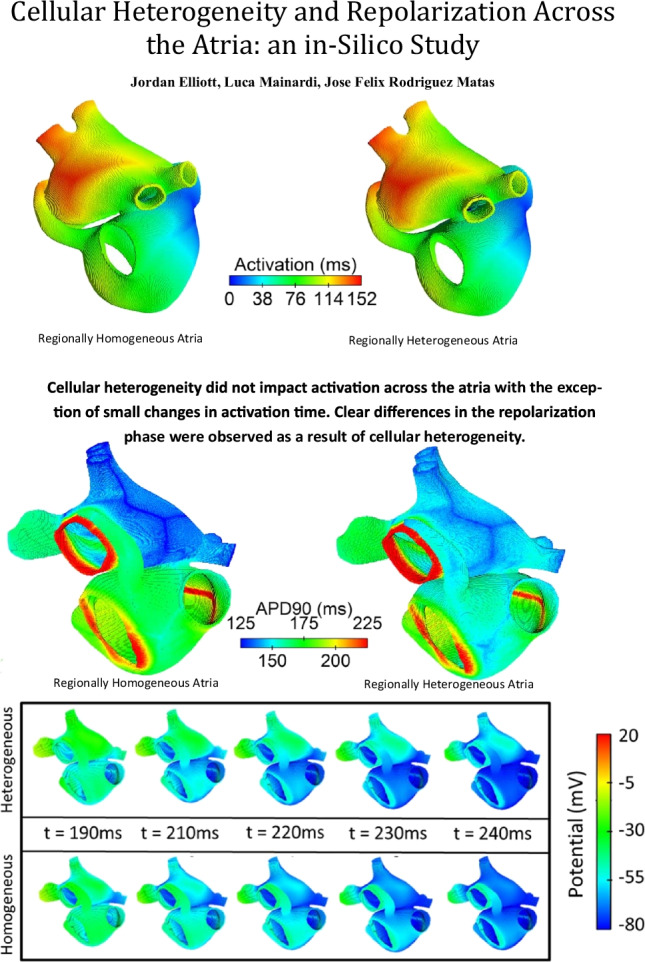

**Supplementary Information:**

The online version contains supplementary material available at 10.1007/s11517-022-02640-x.

## Introduction

Advancements in medical research are driven by the desire to better understand the way in which the human body works in order to better treat patients when the human body does not function as it should. Through better understanding the causes, mechanisms, and impacts of pathophysiological conditions, targeted treatment or prevention methods can be identified and created to improve quality of life and reduce mortality rates associated with these medical conditions. One method for better understanding a particular condition is through in silico modelling. Through the use of mathematical models to represent the mechanisms and behaviours of a biological system on multiple scales, it is possible to further the understanding of how a pathophysiological condition begins, adapts, progresses, and potentially could be reversed. Such information is increasingly valuable when it concerns conditions that impact an increasing number of people around the world. More accurate in silico models inevitably provide more reliable information that better represents the real-life mechanisms of the human body under certain conditions. One such condition that is actively being researched is that of atrial fibrillation.

Atrial fibrillation (AF) is the most common sustained atrial arrhythmia worldwide, typically caused by ectopic beats leading to rapid reentry patterns resulting from unidirectional blocks within the atrial tissue [1]. The mechanisms underlying atrial fibrillation are still not fully understood [2]. The hope is that, with an improved understanding of AF mechanisms, treatment methods can be improved [3]. Characteristic changes to cellular action potentials resulting from electrophysiological remodelling due to chronic or long-standing persistent atrial fibrillation include a more triangular action potential shape [4]; reduced action potential duration (APD); increased APD20 (action potential duration at 20% repolarization) [5–7]; a less pronounced plateau phase [4]; and reduced tissue conduction velocity [8, 9]. Comprehensive and detailed summaries of atrial fibrillation can be found in [4, 10–13]. These changes on a cellular level influence the electrophysiological behaviour on the whole-atrial scale, resulting in increased susceptibility to reentries.

In silico modelling can be useful in understanding the mechanisms of atrial fibrillation and can provide information that is otherwise unobtainable. For this information to be useful, however, it is necessary for the results to be reliable and accurately represent the realistic behaviour in the human atria. This leads to highly complicated multiscale models that incorporate realistic anatomy, fibre direction, material properties, electrophysiological properties, and, in the case of atrial fibrillation, fibrotic tissue. To simplify these already highly complex models, the assumption made is that electrophysiological cellular differences within the same atrial region will be masked due to cellular coupling. Using this assumption, all cells represented in a singular atrial region have identical electrophysiological characteristics. Typically, whole atria studies in atrial fibrillation use this regional homogeneity to reduce the complexity of the models with the assumption that it does not compromise the results [14–19].

Many studies into the mechanisms of atrial fibrillation make this same assumption. Experimental data collected from actual atrial tissue shows variability in electrophysiological properties between cells, including in neighbouring cells [5, 7, 17, 20–26]. Despite this, the assumption remains that the cellular coupling renders these differences negligible, with only a small number of studies considering the impact of cellular variability within atrial tissue [17, 27]. Some investigation into variability has been undertaken on a single cellular basis, using the population of models approach [9, 17, 22, 28–31], but this has not yet been fully utilised in whole atrial simulations.

Atrial fibrillation and the susceptibility to reentries results from the repolarization across the atria. Ectopic beats that cause reentries, and the path those reentries take is defined by the way in which the atria repolarises. This repolarization could potentially be impacted by cellular variability within the tissue. The impact of electrophysiological tissue heterogeneity on the repolarization phase has not yet been established. This paper aims to focus on the repolarization across the atria and the impact of cellular heterogeneity on this phase of atrial behaviour. It is important to determine if cellular variability impacts the repolarization across the atria to a point where there could be significant changes to re-entry patterns or ectopic beat behaviour. This paper presents the impact of introducing cell-to-cell heterogeneity into the AF remodelled whole-atrial model during sinus rhythm. With a particular focus on the repolarization phase across the atria, the behaviour of a regionally homogeneous atrial model is compared with a comparable model that includes cell-to-cell heterogeneity. The outcome of this paper is to determine if the assumption that cellular coupling renders the impact of cellular variability negligible in the overall electrophysiological behaviour of the atria.

## Materials and methods

### Population creation

A non-bias population of 200,000 unique action potential models was created using the Courtemanche–Ramirez–Nattel (CRN) cellular model [32]. To create these unique action potential models, 9 maximum channel conductances: the fast sodium channel conductance, gNa; the transient outward potassium channel conductance, gTo; the ultra-rapid potassium channel conductance, gKur; the rapid delayed rectifying potassium current, gKr, the slow delayed rectifying potassium current, gKs; the time-independent potassium current conductance, gK1; the L-type calcium current conductance, gCaL; the sodium–potassium pump maximum conductance, gNaK; and the sodium-calcium exchanger maximum conductance, gNaCa, were varied between − 100 and + 200% of the standard value, using the Monte Carlo sampling method. This non-bias population included a wide range of maximum channel conductance values and could be calibrated purely on the characteristics of the action potential morphology, without first assuming limitations or characteristics of the channel conductances.

Each unique combination of parameters was stimulated with 101 impulses at a BCL of 1000 ms, with a stimulus duration of 1 ms and amplitude of − 45pA/pF. The action potential resulting from the last impulse was saved and used for classification based on action potential morphology. Following the ultimate action potential, a period of 10 s was recorded with no additional stimulus to determine the stability of each action potential model. Exclusion criteria applied to the total population were used to prevent the inclusion of mathematically unstable or unrealistic action potentials in the populations. Exclusion criteria included any action potentials with spontaneous depolarization activity, a resting membrane potential less negative than − 50 mV, any APD exceeding 1 s, any non-physiological peak voltages, or peak voltages below zero. This prevented unstable, self-exciting or non-physiological action potentials from being included in the population.

MATLAB 2019b (The Mathworks Inc.) was used to both create and stimulate the population and to classify the created populations along with the statistical analysis methods presented. Matlab 2019b was further used for statistical analysis of the results of the whole atrial simulations.

### Regional clustering

**Fig. 1:**
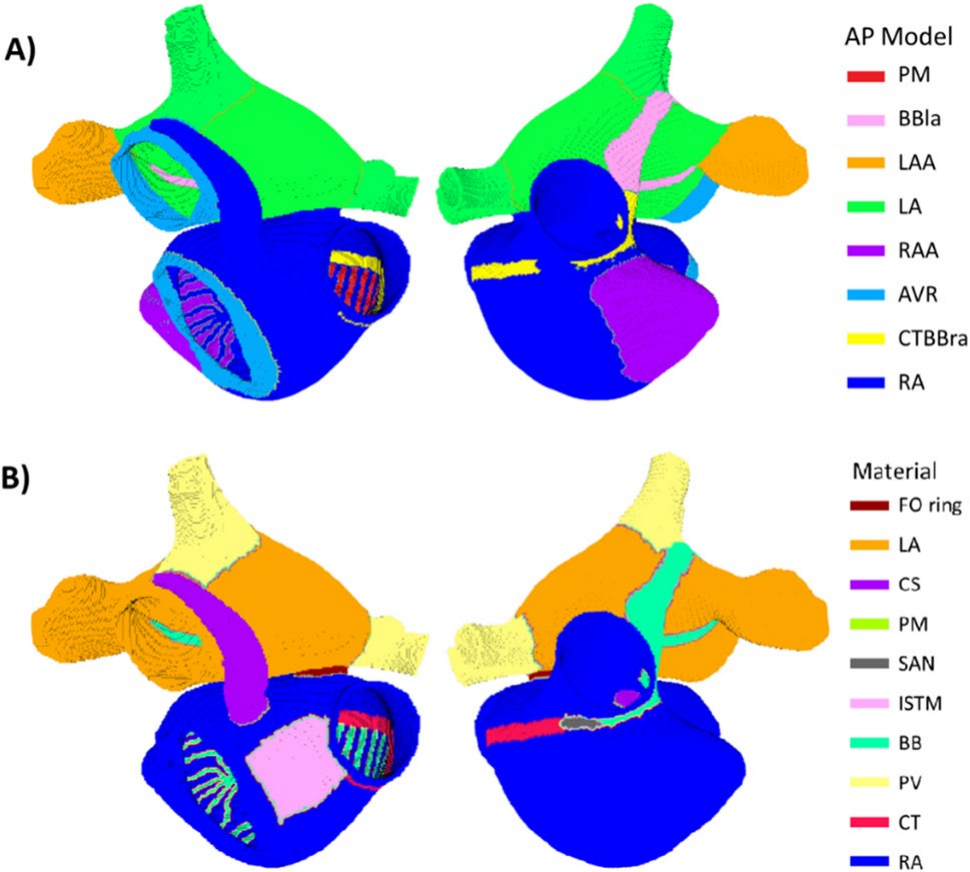
Anatomical atrial model, showing block colours for action potential model assignment in the atria (**A**) and atrial regions based on material properties (**B**)

From the population of 162,971 stable action potential models, smaller populations were extracted based on action potential morphology. Using previously published experimental data [1, 5, 7, 14–16, 20, 23, 26, 30, 31, 33–41], 5 biomarkers were identified for classification. These were the resting membrane potential (RMP), action potential amplitude (APA), and the time at which the potential repolarises 20%, 50%, and 90% (APD20, APD50, and APD90, respectively). According to the characteristics of the action potential, the atria have been divided into eight different electrophysiological regions [8], namely: right atrium (RA), right atrium appendage (RAA), left atrium (LA), left atrium appendage (LAA), atrioventricular ring (AVR), crista terminalis and right Bachmann Bundle (CT/BBra), left Bachmann bundle (BBla), and pectinate muscles (PM). Figure 1A shows the atrial regions to which each cell model has been assigned.

Due to a lack of available experimental data**,** a complete characterisation of regional action potential morphology for the AF remodelled atria does not exist. For the purpose of creating a complete regional classification of AF remodelled tissue, it was assumed that AF remodelling would impact each atrial region in the same way. For example, experimental data suggests AF remodelling results in a reduction of APD90 by approximately 30% in the RAA. The assumption that follows is that all atrial regions would result in a 30% reduction in APD90 as a result of AF remodelling. Further to this, regional APD90 values for healthy tissue in all regions are reported in [35], and the regional differences in APD90 were reported in [8]. Combining these provides a mean APD90 value for AF remodelled tissue in all atrial regions. Similarly, APD50 values in the healthy atrial tissue were combined with the assumption that all atrial regions would result in the same percentage change as a result of AF remodelling. Percentage changes between healthy and AF remodelled tissue were calculated based on data in [39] that reports experimental data for all biomarkers for healthy and chronic AF remodelled tissue for the RAA.

For the APD20, there are decidedly fewer data available, so all regions were set to the same value as given in [5–7]. Due to the method used to characterise the AF remodelled atrial regions, populations were created only for atrial regions where a complete set of biomarker ranges could be characterised. In particular, insufficient data relating to the APD50 for the PV prevented this region from being included.

Table 1 shows the final regional biomarkers mean and standard deviation for regional clustering in the AF remodelled case. Using the mean and two standard deviations for each regional biomarker, the population of models was used to create regionally specific populations, including all action potential models that satisfied the criteria for all biomarkers. The full process of creating the population of models and the classification of regional biomarkers is outlined in [29].

**Table 1:** Experimental (top) and simulated populations (bottom) mean and standard deviation of the AF remodelled biomarkers for each of the eight defined atrial regions, used to create regional populations. For the purpose of clustering, regional populations included the mean ± 2 standard deviations. This data is based on combined experimental data collated from multiple publications. The bottom half of the table shows the mean and standard deviation achieved for each region using the population of models approach and clustering based on the experimental values shown in the upper part of the table

		RA	RAA	LA	LAA	AVR	CT/BBra	BBla	PM
Experimental	RMP	− 78 ± 12	− 79 ± 6.6	− 78 ± 5.4	− 73.8 ± 6.6	− 73.8 ± 1.4	− 77 ± 1.9	− 77 ± 1.9	− 75.9 ± 12
	APA	116.6 ± 14	124.1 ± 19	112.4 ± 13	128 ± 19	127.3 ± 21	134.8 ± 19	124.1 ± 19	131.6 ± 16
	APD20	30 ± 18	30 ± 18	30 ± 18	30 ± 18	30 ± 18	30 ± 18	30 ± 18	30 ± 18
	APD50	72.2 ± 37	105.6 ± 36	54.7 ± 17	89.7 ± 13	38 ± 21	119.3 ± 32	94.2 ± 32	74.5 ± 17
	APD90	200 ± 62	190 ± 22	174 ± 34	160 ± 22	170 ± 29	219 ± 64	172 ± 32	172 ± 19
*Population*	*RMP*	− *83.5* ± *2.4*	− *83.9* ± *2.0*	− *83.7* ± *1.9*	− *84.4* ± *1.6*	− *74.5* ± *1.5*	− *78.6* ± *2.0*	− *78.5* ± *1.6*	− *84.0* ± *1.6*
	*APA*	*119.9* ± *13.2*	*119.1* ± *13.6*	*117.5* ± *13.1*	*118.9* ± *13.5*	*115.2* ± *12.8*	*119.6* ± *10.4*	*118.6* ± *10.8*	*121.7* ± *10.8*
	*APD20*	*5.6* ± *7.9*	*8.89* ± *11.6*	*4.5* ± *3.8*	*8.4* ± *9.4*	*4.1* ± *4.4*	*8.6* ± *11.0*	*10.1* ± *6.4*	*6.0* ± *6.4*
	*APD50*	*62.3* ± *43.9*	*109.4* ± *35.2*	*53.6* ± *20.1*	*90.0* ± *14.9*	*28.0* ± *27.8*	*121.5* ± *34.2*	*86.3* ± *19.5*	*79.1* ± *19.5*
	*APD90*	*163.0* ± *59.9*	*188.7* ± *24.6*	*151.7* ± *34.1*	*158.5* ± *22.3*	*205.2* ± *60.5*	*271.1* ± *51.3*	*197.8* ± *28.1*	*164.3* ± *20.6*

The atrial model was divided into ten anatomical regions with different conductance and fibre orientation [36]: right atria (RA), crista terminalis (CT), pulmonary veins (PV), Bachmann bundle and pectinate muscles (BB/PM), isthmus (ISTMO), sinoatrial node (SAN), fossa ovalis (FP), fossa ovalis ring (FO ring), coronary sinus (CS), and left atrium (LA). Figure 1B shows the different anatomical regions based on material properties. Each of the anatomical regions can host cells with different action potential models. Table 2 shows the content of each tissue material based on the action potential models.

**Table 2:** Action potential model content of atrial tissue materials

Tissue	Cell models present
RA	RA (80.0%), AVR (7.9%), RAA (12.1%)
CT	RA (24.6%), CT/BBr (73.3%), PM (2.1%)
PV	RA (0.1%), LA + PV (99.9%)
BB/PM	RA (44.0%), CT/BBr (9.8%), LA + PV (5.1%), BBl (21.6%), PM (19.5%)
ISTMO	RA (100.0%)
SAN	RA (97.6%), PM (2.5%)
FO	CV = 0
CS	RA (100.0%)
LA	RA (0.4%), CT/BBr (12.0%), LA + PV (69.7%), LAA (18.0%)
FO ring	RA (100.0%)

Using the regional populations, ten unique atrial models including cellular variability were created for the AF remodelled case. Through random, uniform distribution, each node in an atrial region was assigned one of the action potential models from the regional population of models. The regionally homogeneous model was created by assigning the regional population mean action potential model to each node assigned to that atrial region. Nodes in the PV regions in the atrial model were assigned AP models from the LA population. Whereas experimental data shows that the APD90 of the PV is 10–15% reduced compared with the LA [42] in healthy tissue, insufficient data for the other biomarkers prevented the complete characterisation and creation of this population.

Further to this, due to the location of the PV in the atria, the use of the LA population in the PV nodes has little impact on the total depolarization and repolarization across the atria. The PV is typically one of the last areas of atrial tissue to depolarise so it was decided that the use of the LA population for the PV nodes would have a negligible impact on results. This use of the LA population in the PV nodes is more significant when looking at the reentrant activity and ectopic beats occurring around the PV. Other previous studies have also not separated LA and PV regions with regards to underlying action potential models [18, 19, 39, 43, 44].

The AP of SAN cells were not modelled, and therefore their self-stimulating characteristics are not present in the whole atrial model. An external stimulation was provided at the location of the SA node, as has been performed in a number of previous studies dealing with atrial fibrillation [8, 16]. Due to this limitation, the SAN region was excluded from the analysis. Furthermore, when computing the results, boundary elements from the SA node were removed (three layers) in order to minimise the possible effect of electrotonic coupling associated with the external stimulation applied to the model.

Furthermore, nodes occurring on a direct boundary and that being an element within 3 layers of a direct regional boundary were removed from analysis in order to minimise the possible effect of electrotonic coupling associated with the external stimulation applied to the model. This was also to remove any potential unequal/regionally bias in distortion of variability in the atrial regions due to varying proportions of boundary nodes with respect to middle (or non-boundary) nodes. For example, the PM region had a much larger ratio of boundary nodes to middle nodes than the RA or LA regions. Not removing these boundary nodes from analysis would result in an artificially increased degree of variability in the PM region due to boundary node distortion. The removal of these nodes from the analysis could impact the results. However, not removing these nodes would result in skewed results in the smaller regions with increased boundary node to middle node ratios.

### Whole atrial simulation

Electric propagation in the atria was model through the monodomain model

1 $$\begin{array}{ccc}{C}_{m}\frac{\partial V}{\partial t}=\nabla \bullet \left({\varvec{D}}\bullet \nabla V\right)-{J}_{ion}\left(\mathbf{s},V,t\right)& \mathrm{in}& H,\\ \frac{\partial \mathbf{s}}{\partial t}=\mathbf{f}\left(\mathbf{s},V\right)& \mathrm{in}& H,\\ {\varvec{n}}\bullet \left({\varvec{D}}\bullet \nabla V\right)=0& \mathrm{in}& \partial H,\end{array}$$

where $V$ is the transmembrane potential, ${J}_{ion}\left(\mathbf{s},V,t\right)$ is the ionic current associated with AP model i.e., the CRN model, ${\varvec{s}}$ are the state variables of the model, *H* refers to the full atrial tissue domain, $\partial H$ its outer surface and ${\varvec{n}}$ its outer normal, and ${\varvec{D}}$ the conductivity tensor of the tissue assumed as transversely isotropic

2 $${\varvec{D}}={\sigma }_{L}\left[\left(1-r\right){{\varvec{n}}}_{f}{{\varvec{n}}}_{f}^{T}+r{\varvec{I}}\right],$$

with ${\sigma }_{L}$ the conductance along the fibre, ${{\varvec{n}}}_{f}$ the fibre direction, $r={\sigma }_{T}/{\sigma }_{L}$ the transverse to longitudinal conductivity ratio, and ***I*** the identity matrix.

The monodomain model Eqs. (1) and (2) was solved by means of the finite element method in combination with operator splitting numerical scheme as described in [12] geometry of the atria having a wall thickness between 600 and 900 μm, was discretised with trilinear hexahedral elements with a regular space resolution of 300 μm, resulting in a mesh with 754.893 nodes and 515.010 elements. This element size was chosen as a good compromise between computing efficiency and adequate spatial resolution to capture the depolarization phase of the action potential in the different regions of the atria. The reader is referred to [8] for additional details of the model. The values of ${\sigma }_{L}$ and ${\sigma }_{T}$ were obtained through calibration of the conduction velocity in each region of the atria. To do this, using the same AP model content, in a slab model (1.8 × 1.8 × 18 mm^3^) meshed with trilinear hexahedra with the same spatial resolution as the atrial model, 300 μm isolated tissue samples were created for each anatomical region. The target transverse and longitudinal CV for the homogeneous and heterogeneous tissue samples can be found in Table 3.

**Table 3:** Target conduction transverse and longitudinal conduction velocity values for the AF remodelled material regions

Region	Target longitudinal conduction velocity (cm/s)	Target transverse conduction velocity (cm/s)	Region	Target longitudinal conduction velocity (cm/s)	Target transverse conduction velocity (cm/s)
RA	78.51	30.52	SAN	23.29	23.29
CT	86.52	33.66	FO	0	0
PV	58.64	43.98	CS	92.82	58.11
BB/PM	100.12	39.21	LA	62.99	30.72
IST	66.27	66.27	FO RING	85.00	33.11

Using the initial conditions for the final stimulus of the single cellular simulations in creating the population of models (POM), a checkpoint file was created to set the initial conditions for each atrial model. The checkpoint file ensured each node in the atrial model had stabilised initial conditions prior to whole atria simulations start. To further stabilise each whole atrial model as a single unit, 10 stimuli were initiated in the SA nodes, at a BCL of 800 ms, with an amplitude of 50 mV and stimulus duration of 2 ms. At the end of this pre-pacing, a checkpoint file was created for the initial conditions of further stimulations and analysis. After pre-pacing, a single stimulus (50 mV, duration 2 ms) was applied to the SA nodes in each atrial model and the resulting propagation was saved and analysed at high resolution. Further to this, the activation time, APD90, and total repolarization time for each node was calculated and maps showing each of these were created for each atrial model. Potential maps at a frame rate of 1 ms were also saved and used to construct videos of the propagation across each atrial model. The mono-domain formulation (Eqs. (1) and (2)) was solved with ELVIRA software [12] a constant time step d*t* = 0.02 ms. Simulation of 8 s of atrial activity took 23 h on a 16-core Intel Xeon 2.9 GHz and 64 GB RAM computing node.

### Impact of electrotonic coupling

Post-simulation analysis of the heterogeneous atria was undertaken using a region-by-region approach to determine the impact of electrotonic coupling. Prior to regional analysis, all nodes associated with elements on regional boundaries were removed to minimise distortion resulting from regional boundaries. Further to this, nodes surrounding nonconductive tissue (FO, FO ring, central column of the BB) and SA node were isolated and removed from the analysis. This was applied to nodes in three layers of elements surrounding these regions. The reason for this was to minimise the impact of these regions from influencing the variability in the atrial models due to distortion resulting from nonconductive tissue and high amplitude action potentials from the initial stimulus. Once these nodes were removed, the atrial nodes were divided into groups based on both regional classification and the action potential model. Therefore, all nodes in a single group consisted of the same material characteristics and action potential models from a single regional population.

The APA, RMP, and APD at 20% (APD20), 50% (APD50), and 90% (APD90) repolarization were calculated for each node within the regional group. Comparing the mean and standard deviation of these groups in the atrial model with that of the populations used to create the heterogeneous shows the impact of electrotonic coupling in the heterogeneous tissue. Further to this, the triangulation of action potentials, i.e., TRI = APD90-APD50, in the population of models was compared with that of the action potentials in the regional groups. With triangulation being an indicator of arrhythmia vulnerability [45], comparing the mean triangulation in the isolated cellular populations with that of the nodes within each region in the atrial model, the impact of electrotonic coupling on susceptibility to arrhythmia could be quantified.

## Results

### Population of models

Clustering action potential models based on the biomarkers set based on experimental data resulted in population sizes ranging from 1193 to 82,025 unique action potentials. The AVR, CTBBr, and BBl regions were the smallest, with the RA and RAA regions being the largest population sizes. Table 4 shows the regional population sizes used to generate the atrial models.

**Table 4:** Regional population sizes after clustering based on biomarkers for the AF remodelled atria

Region	Population size
AVR	1193
BBla	1499
CTBBra	4075
LA	24,427
LAA	15,317
PM	13,612
RA	82,025
RAA	32,979

The mean and standard deviation of regional populations after clustering is presented in the lower half of Table 1. The mean and standard deviation of each regional population differed from the experimental data used to create the populations. The RMP across all regions was more negative in the populations compared with the set values, and the standard deviation in all regions remained within ± 2.0 mV, showing a smaller variability within the POM than observed in experimental data. The APA in the populations also showed less variability across atrial regions than the target values and smaller standard deviations within each regional population.

The APD20 biomarker within each regional population was significantly reduced compared with the target APD20. This is consistent across all regions, with the appendages, crista terminalis, and Bachmann’s bundle managing the largest mean APD20 values. Additionally, there is a reduction in variability in APD20 compared with the target population.

The standard deviation in the APD90 biomarker across all regions successfully matched the target standard deviations. All regions except the LAA showed a drop in the mean APD90. Despite this, the mean for the APD50 biomarker was maintained across most atrial regions, with a reduction of 8–10 ms in the RA, AVR, and BB regions. Again, across all regions, the standard deviation in the APD50 was maintained, with a slight increase in the RA, LA and AVR, and a reduction in the BBla region.

**Fig. 2:**
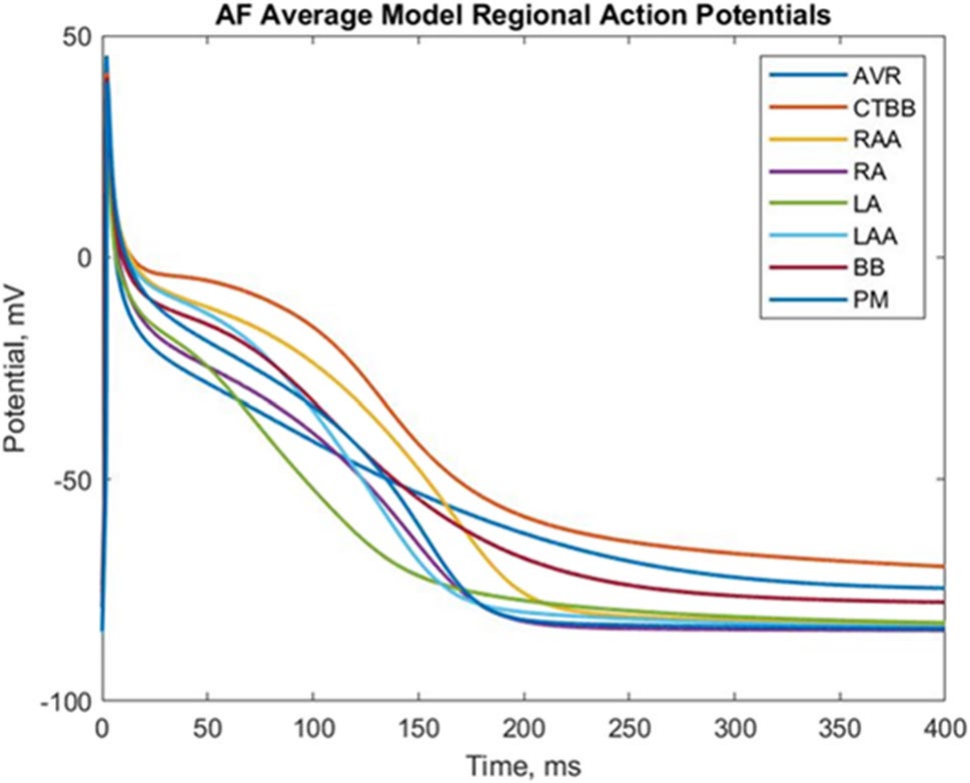
Mean action potential models for each regional population for the AF remodelled atrial regions

Figure 2 shows the mean action potential model for each atrial region based on the five biomarkers used for classification. These action potential models were used for the regionally homogeneous atrial models for comparison with the heterogeneous atrial models.

### Isolated tissue samples

**Fig. 3:**
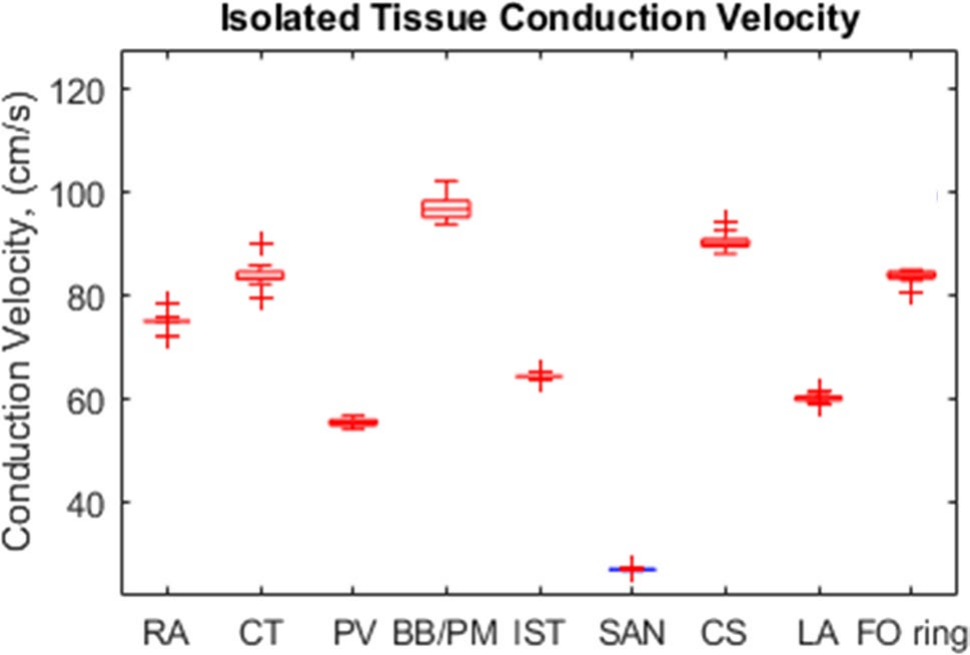
Boxplot showing the tissue material CV across 10 heterogeneous AF remodelled tissue samples for each region

Figure 3 shows the variability in conduction velocity for each tissue material across 10 unique tissue samples for each material used for calibration. The conduction velocity of the fossa ovalis tissue, not shown in the figure, was set to 0 cm/s. The highest degree of variability was observed in the CT and BB/PM tissues, with a standard deviation of 2.7 and 2.4 cm/s, respectively. The SA node showed the least variation, with a standard deviation of 0.1 cm/s, followed by the isthmus with a standard deviation of 0.39 cm/s. The regions with the two largest areas in the atrial model and therefore would have the greatest impact on the whole atrial model were the RA and LA. The standard deviation in conduction velocity across the 10 tissue models in these tissues were 1.4 and 0.85 cm/s, respectively.

### Whole atrial simulations

**Fig. 4:**
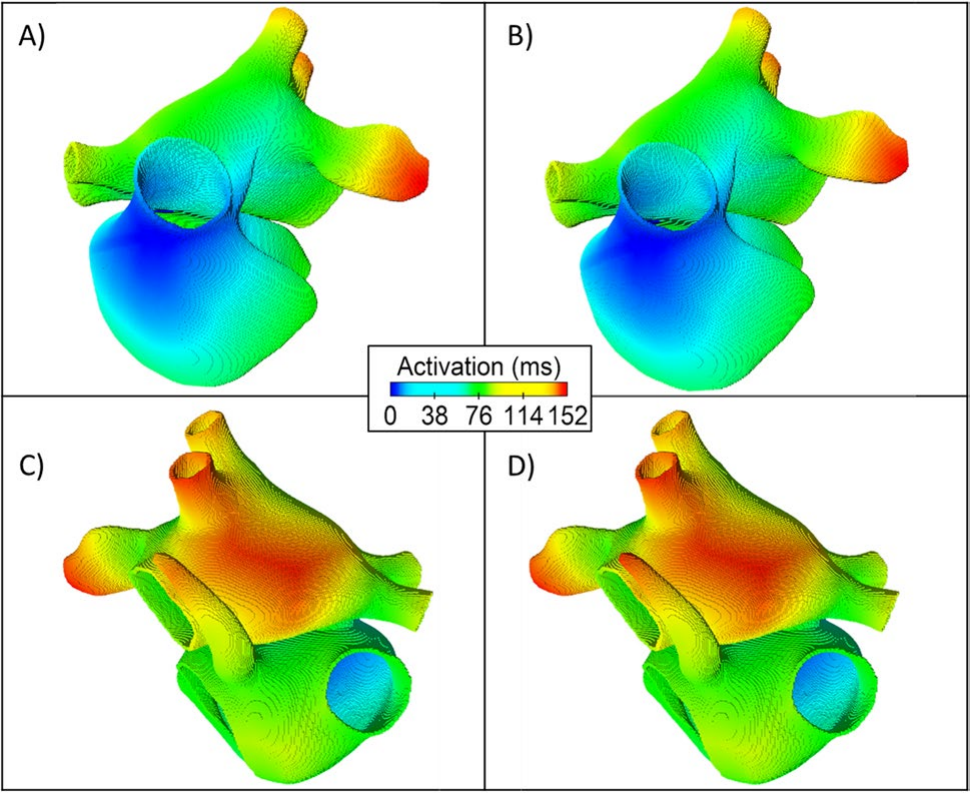
Activation maps for the homogeneous (**A** and **C**) and heterogeneous (**B** and **D**) atria. The centre of the figure shows the colour scale for the activation time

There was no significant difference in the activation across the atria resulting from cellular heterogeneity. Figure 4 shows the activation maps across the homogeneous (Fig. 4a and c) and heterogeneous (Fig. 4b and d) atrial models. The total activation time in the homogeneous atrial model was 146 ms and the total activation time was 147 ms across all heterogeneous atrial models.

**Fig. 5:**
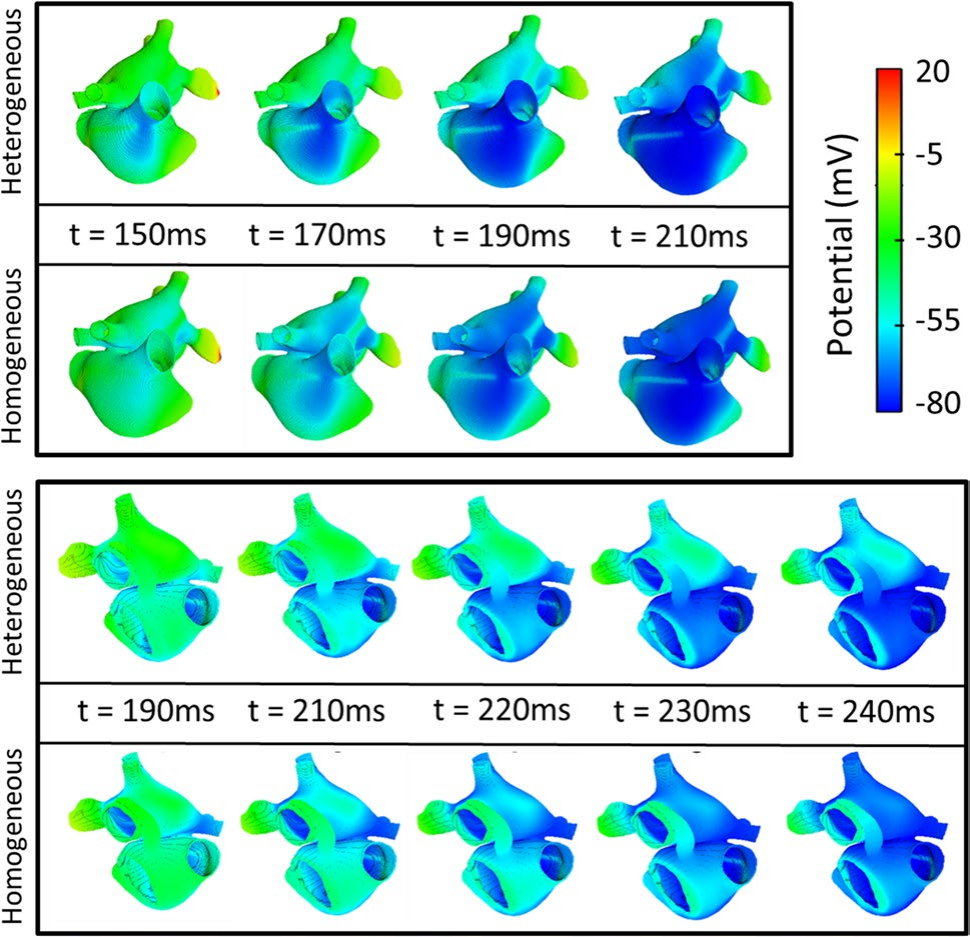
Repolarization across the atria over time. **A** The repolarization from an anterior position with the left atria superior to the right atria. **B** The posterior view of the atria, showing the left atria superior to the right atria. In both cases, the heterogeneous or variable model is presented above the associated time frame, and the equivalent regionally homogeneous or average model is presented below the timeframe

However, the repolarization across the atria varied between the homogeneous and heterogeneous atrial models. The repolarization patterns remained consistent across the 10 heterogeneous models. This suggests the visual difference was not specific to a single variable model but was a result of the intrinsic cellular heterogeneity. Figure 5 shows the repolarization across homogeneous and heterogeneous atrial models from 150 to 240 ms after initially stimulating the sinoatrial node. Showing the repolarization across the atria from anterior and posterior viewpoints. This figure clearly highlights differences in repolarization as a result of cellular heterogeneity. The LA region repolarises quicker in the homogeneous atria than in the heterogeneous atria, whereas the reverse is observed in the right atria.

**Fig. 6:**
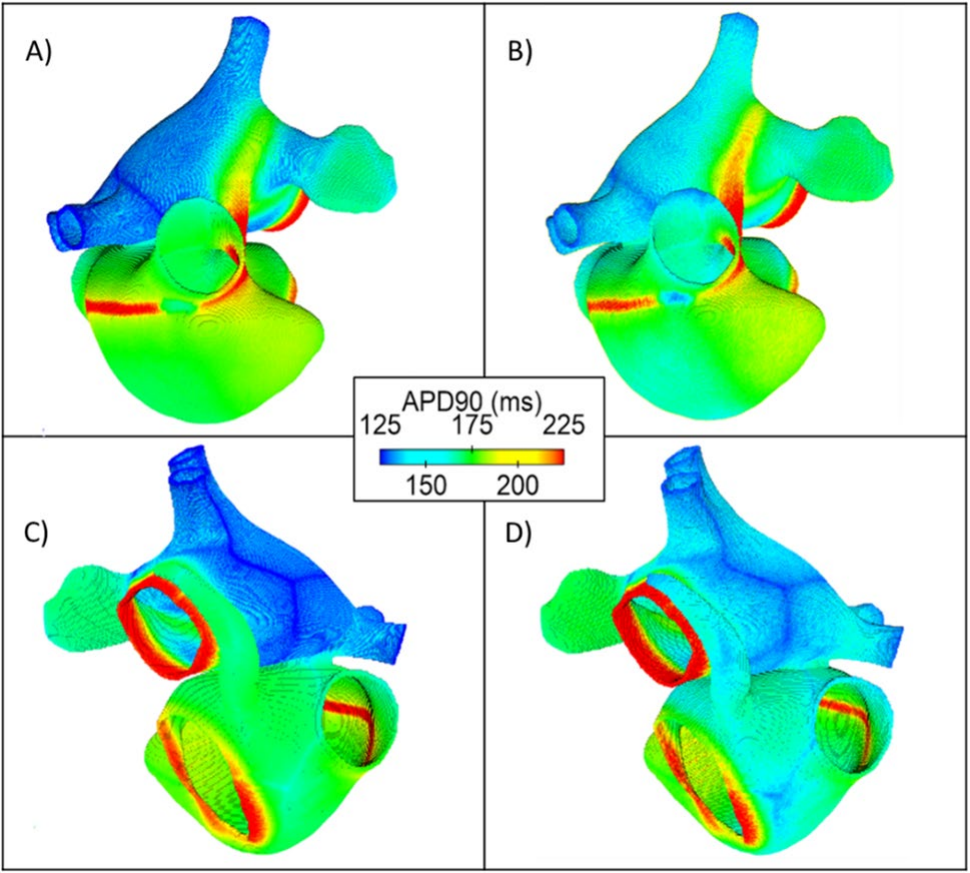
APD90 maps for the homogeneous (**A**, **C**) and heterogeneous (**B**, **D**) atrial models. The centre of the figure shows the colour scale for the APD90

The APD90 of nodes across the atria, shown in Fig. 6, further confirms this. In Fig. 6a and c, the homogeneous atrial model shows that APD90 across the LA region is reduced compared with the heterogeneous atrial model. As can be observed in both Figs. 5 and 6, the impact of cellular heterogeneity on repolarization differs from region to region. Further to this, the APD90 shows not only regional differences as a result of cellular heterogeneity but also shows variability in APD90 across individual atrial regions.

**Fig. 7:**
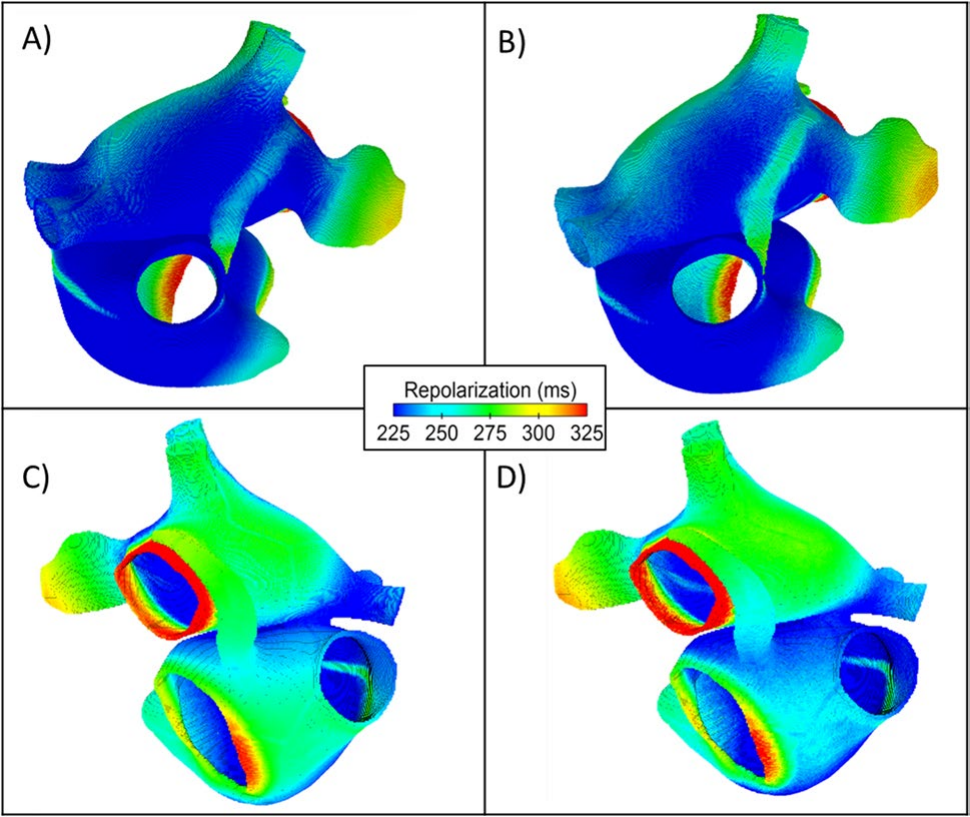
Total repolarization time maps for the average (**A**, **C**) and variable (**B**, **D**) atrial models. The centre of the figure shows the colour scale for the repolarization time, calculated by combining the activation time with the APD90 for each node

Combining the activation time with the APD90 gives the total repolarization time for each atrial node. Figure 7 shows the total repolarization maps for the homogeneous (Fig. 7a and c) and heterogeneous (Fig. 7b and d) atrial models. Again, all 10 heterogeneous atrial models showed similar repolarization maps. Whereas the differences between homogeneous and heterogeneous models are less distinguishable in the total repolarization maps, some characteristics showed clear differences.

Figure 7b shows a clearer boundary between the BB region and the surrounding LA region than the equivalent region in the homogeneous model, Fig. 7a. Additionally, heterogeneity results in a reduction in repolarization time across the right atria, showing as a light blue in the posterior view of the atria in Fig. 7d compared with the homogenous model in 7c, showing a green region and therefore increased repolarization time. The AVR region in the homogeneous and heterogeneous atrial models show no observable differences.

### Electrotonic coupling impact

**Fig. 8:**
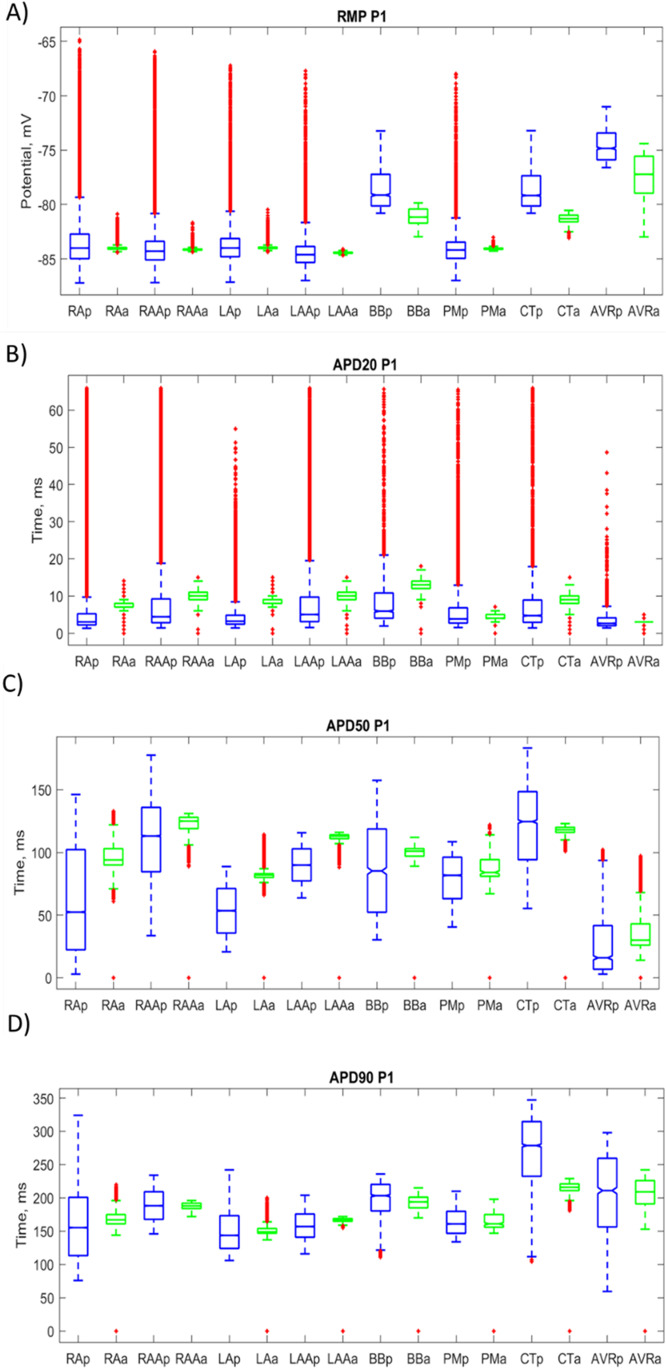
Boxplots showing the distribution of biomarkers RMP, APD20, APD50, and APD90 across regional population of models (blue) and regions in the whole atrial simulations (green)

To determine the impact of electrotonic coupling on the variability in the atrial model, the action potential characteristics within the regional atrial tissue were compared with that of the populations used to create them. The RMP and APD20, APD50, and APD90 repolarization were calculated for each node within the regional tissue in one of the heterogeneous patient atrial models (similar behaviour was observed in all heterogeneous models). Due to the sampling rate in the whole atrial simulations and the nature of the initial phase of the action potential, the APA biomarker was deemed unreliable and therefore excluded from the comparison. The distribution of these biomarkers across each region in the whole atrial simulations was compared with the distribution in the regional population of models. Figure 8 shows the distribution of the biomarkers for each regional population in blue and nodes across each region in the heterogeneous atrial model in green.

In all regions, there is a substantial reduction in variability for all biomarkers, with the exception of the RMP in the AVR region. Electrotonic coupling causes a reduction in RMP variability across all atrial regions except the AVR. The variability in the AVR actually increased 28.6%, from 1.5 to 1.95 mV. In the remaining regions, the RMP standard deviation was reduced between 64 and 95%. For the CTBBr, BBl, and AVR, there is also a reduction in the mean RMP value (3.5%, 3.3%, and 3.9%, respectively). The remaining regions showed consistency in the mean RMP.

An increase in the mean APD20 was observed across all regions except the AVR and PM. This increase in APD20 brings the raw value in APD20 in line with experimental data for APD20 in AF remodelled tissue. Whereas the Courtemanche cellular model in the single cellular simulations was unable to create populations with APD20 values in experimental data, the whole atrial simulations increased the APD20 of cells to experimental ranges. The largest increase in the mean APD20 was observed in the LA, with an increase of 98% from 4.5 to 8.9 ms. Furthermore, the standard deviation in APD20 was reduced in all regions by 72–88%, with the largest reduction occurring in the RAA and the smallest reduction occurring in the LA.

For the APD50 biomarker, a reduction in the standard deviation was observed across all regions of between 33% (in the PM) and 86.5% (in the BB). Interestingly, the mean APD50 was increased in all atrial regions except the CT region, which reduced APD50 by 3.6% in the atrial simulations compared with the population of models. The largest increase in APD50 was observed in the AVR region, with a 75.7% increase. This, in part, was due to the AVR population having the smallest APD50 values of all regions. The RA and LA regions also showed an increase in APD50 of 55 and 54%, respectively. Again, these regions had smaller APD50 values in the population of models.

Again, a significant reduction in the variability of APD90 was observed across all atrial regions. The standard deviation was reduced by between 24.8% in the PM (from 20.6 to 15.5 ms) and 83% in the LAA (from 22.3 to 3.8 ms). In the atrial simulations, the region with the smallest standard deviation in APD90 was the LAA, and the region with the largest was the AVR, with a standard deviation of 21.7 ms. The mean APD90 between populations and atrial regional tissue remained fairly consistent. The CT region showed the greatest change in APD90 due to cellular interaction and electrotonic coupling. The CT population had a mean APD90 of 271 ms, whereas, in the CT region of the whole atrial simulations, the mean APD90 was reduced to 214 ms. In both cases, the CT region had the largest mean APD90 across all regions. Other atrial regions showed a negligible change in the mean APD90 value of between a 3% reduction and a 5% increase.

### Triangulation

Through the inclusion of cellular heterogeneity, there is a marked reduction in the triangulation, as shown in Table 5. The table shows that triangulation in the LA region was reduced from 89 to 69 ms as a result of heterogeneity. This behaviour was observed in lesser or greater effect in all regions, except for the AVR region, where an increase in triangulation as a result of heterogeneity was observed. There is also a significant reduction in the triangulation between the single cell model and the full 3D atria (tissue) model due to electrotonic coupling (see Table 5). As expected, the variability is also significantly reduced in this case.

**Table 5:** Effect of coupling on triangulation. Columns 2 and 3 show the mean triangulation (ms) in regions for the homogeneous and heterogeneous atrial models. Columns 4 and 5 show percentage change in mean and standard deviation of triangulation resulting from electrotonic coupling. Percentage changes were calculated from the single cell populations to regions in the whole atrial model

	Effect of variability (homogeneous versus variable 3D model) Mean regional triangulation (ms)		Effect electrotonic coupling (3D versus single cell population)	
	Homogeneous atrial model	Heterogeneous atrial model	% change in mean	% change in SD
RA	77	72	− 28	− 83
RAA	70	65	− 18	− 80
LA	89	69	− 29	− 81
LAA	62	55	− 19	− 70
AVR	156	172	− 2.9	− 45
BB	95	93	− 17	− 71
CT	108	98	− 35	− 78
PM	85	76	− 11	− 56

## Discussion

No significant difference was observed in activation time between regionally homogeneous and heterogeneous models. With total activation time being strongly related to tissue conduction velocity, the lack of variability in total activation time is not surprising. The RA and LA are the two largest regions in the atrial model and likely have the most significant impact on the total activation time in the atria. The variability in conduction velocity across the 10 models used for calibration were 1.45 and 0.85 cm/s, respectively. This small variability in the conduction velocity across the 10 heterogeneous models would suggest the activation times across multiple heterogeneous models should be consistent. This is confirmed in the whole atrial simulation. Furthermore, because the isolated homogeneous and heterogeneous tissue samples were independently calibrated to match conduction velocity, the depolarization across the homogeneous and heterogeneous atrial models should be similar. However, the value of the calibrated conductances for homogeneous and heterogeneous models differed by less than 5% in all cases. This indicates that eventual differences in the AP upstroke are smeared out by electrotonic coupling and that the variability in atrial activation times reported in the literature [46] is mostly due to anatomical differences between patients rather than the intrinsic electrophysiological variability at the cell level.

Previous studies show action potential morphology is influenced by the frequency of stimulation [5, 31, 36]. It could then be argued that the change in BCL from 1000 to 800 ms when moving from the single cellular model to the whole atrial model would result in significant changes in the atrial behaviour due to deviation from the steady state.

It was decided to use a BCL of 800 ms as this cycle length is more representative of normal heart rate. This change in BCL from the single cellular model to the whole atrial model would likely result in the atrial model not reaching a steady state. Restitution curves for the APD90 in the Courtemanche cellular model were calculated. This restitution curve is presented in Supplementary material in Fig. S1. By reducing the BCL to 800 from 1000 ms, APD90 was reduced by 0.69%. To determine the impact of this change in BCL, the atrial model was run at BCL = 1000 ms and BCL = 800 ms, and the activation, APD90, and repolarization were compared on a regional basis. Boxplots showing the regional distribution of the activation, APD90, and repolarization are included in Supplementary material in Fig. S2.

Across all regions, there were insignificant changes to the mean and standard deviation of the activation. The largest regional change in mean activation time was 0.33% in the average model and 0.33% in the variable model. The largest regional change in the standard deviation of the activation time was 0.47% in the average model and 0.37% in the variable model.

The largest regional change in mean repolarization time was 1.83% in the average model and 2.7% in the variable model. The mean regional change in standard deviation of the repolarization time was 0.6% in the average model and 0.93% in the variable model.

The largest regional change in mean APD90 time was 2.32% in the average model and 3.0% in the variable model. The mean regional change in standard deviation of the repolarization time was 2.44% in the average model and 2.33% in the variable model.

These small changes in the mean and standard deviations of the activation time, repolarization time, and APD90 may confound observations, but only to a small extent, and therefore observations in this study are not significantly impacted by the change in the BCL. Overall, cellular heterogeneity impacted the repolarization of each atrial region to a different degree. For example, the LA region showed prolonged repolarization time in the heterogeneous model, whereas in the RA there was a reduction in repolarization time, as shown in Fig. 5. The impact of heterogeneity in each region was repeated across all 10 heterogeneous atrial models, whereby the experimental protocol remained the same but the specific node assignment was unique to each model. This would suggest that the results observed are a result of the overall variability in the tissue rather than being unique to a specific case. One observation was the increase in repolarization time in the LA region as a result of heterogeneity. This was due to an increase in the mean APD90 for this region, from 152 to 159 ms. Unlike the LA region, the RA region repolarised faster in the heterogeneous atria. This was due to a reduction in the regional mean APD90 from 179 to 169 ms. Unfortunately, no other papers have previously reported the impact of cellular heterogeneity on the APD90 from region to region. The more pronounced boundary between the BB and LA regions in the heterogeneous model could result in an increased window of vulnerability for unidirectional propagation and, therefore, reentry.

It is widely understood that cellular electrotonic coupling within atrial tissue results in a reduction in variability [39]. Further to this, it is also understood that some diseases cause an uncoupling between cells, and therefore an increase in cellular variability is to be expected in unhealthy tissue [22, 39]. It is therefore particularly important to determine the impact of cellular heterogeneity within the atria in such conditions. Despite this, few studies look at the impact of cellular heterogeneity within atrial models, and none, to the authors’ knowledge, investigate the impact of electrotonic coupling on the cellular variability in tissue or whole atrial models. In this study, almost all regions showed a reduction in the variability of all biomarkers. This shows that electrotonic coupling does significantly reduce variability, as reported in the literature [22] and is observed experimentally [5, 21]. Electrotonic coupling is unable to completely remove variability, however, and results in different repolarization across the atria. Furthermore, the variability in the tissue samples used for calibration for these regions possibly contributes to the variability observed in the whole atrial model, but the variability in the tissue simulations is smaller than the variability in the respective whole atrial regions, so this cannot be the main cause of variability in the atrial model regions.

The variability in the RMP biomarker in the AVR is increased despite electrotonic coupling. This shows regional boundaries have a strong impact on variability in spite of electrotonic coupling. Similarly, in the CT and BB regions, variability in RMP is reduced, but to a lesser extent than in the larger atrial regions. Again, this shows the action potential variability in smaller regions is impacted by neighbouring regions. The method in this study removed boundary elements from analysis for the impact of electrotonic coupling. The results, therefore, show that the impact of neighbouring boundaries influences not only immediate boundary cells but further into the regional tissues. In [5], there is also a significant reduction in the variability in RMP in the trabeculae compared with the isolated cells. The mean RMP values in the CT, BB, and AVR regions are significantly reduced. The CTBBr, BBl, and AVR are the smallest regions and have a higher percentage of nodes in close proximity to other atrial regions. These other regions have lower mean RMP values. The change in RMP for the smaller atrial regions suggests the electrotonic coupling between neighbouring regions with differing electrophysiological characteristics can have a significant impact on the characteristics of smaller atrial regions. Additionally, considering the elements immediately neighbouring the boundary of each region were removed from the analysis, this shows that the impact extends beyond regional boundaries and can cause a change in the AP morphology and therefore cellular behaviour, with the mean RMP moving closer to that of surrounding regions. In both [5] and [21], RMP is more negative in trabeculae than single cells in the RAA and RA regions, respectively. Interestingly, in [21], the LA region is less negative in the trabeculae than in isolated cells. This could indicate that electrotonic coupling could have an impact on the mean RMP values for atrial regions, which is consistent with the observations in this study for the CT, BB, and AVR regions.

The mean APD50 was increased in all regions except CT. This increase in the mean APD50 from single cells to whole atrial simulations is consistent with the difference in mean APD50 between isolated cells and trabeculae in [5], whereby APD50 is 47 ms larger in the trabeculae and in [21] for both the LA and RA regions. In the CT region, the mean APD50 is reduced due to the properties of the surrounding tissue. Because the RA regional APD50 is significantly smaller than the CT region, electrotonic coupling causes the characteristics of the CT region to be significantly altered. Because of the proportion of boundary cells to middle cells in the CT, this impact of electrotonic coupling is clearer than in other regions. Though no studies explicitly investigate the impact of electrotonic coupling, some studies report experimental observations for both isolated cells and cellular trabeculae or a small group of cells. These studies show a reduction in variability of some biomarkers in the trabeculae compared with the isolated cells. This paper further confirms these changes in variability due to electrotonic coupling. In both this study and experimentally in [5] the variability in APD20 is significantly reduced compared with single cell observations [7, 17]. In this study, the same pattern in reduction in variability as a result of cellular coupling is observed. Additionally, in [31] it is observed that the single cellular Courtemanche models are unable to create APD20 values that reflect experimental data. This study observed the same limitation in the Courtemanche cellular model, but in the whole atrial model, there was a significant increase in the mean APD20 across all regions except the AVR.

Experimental data in [5] shows a reduction in the RA region from single cellular observations to trabeculae. However, it should be noted that the rate of stimulation was at 1 Hz for the single cells and 2 Hz for the trabeculae, and this could impact results. Further to this, it should be noted that these results are in contradiction with [21], where the LA shows a marked reduction in the APD90 (12 ms) but an increase in the APD90 (of 3 ms) for the RA trabeculae compared with the single cellular observations. This could be due to differences in experimental protocol confounding results.

Furthermore, in [5], the standard deviation is reduced in trabeculae compared with isolated cells. This is once again distinct from [21], whereby variability is increased in multicellular preparations compared with isolated cardiomyocytes. Again, this could have been impacted by the difference in stimulation rate. To this effect, [31] reports the experimental data for the RAA single cells at both 1 and 2 Hz stimulus frequency. It showed a 3% reduction in standard deviation due to the increase in frequency and a 1.6% reduction in the mean APD90 due to the increased stimulus frequency. It would therefore be reasonable to assume that the impact of change in frequency of stimulation is minimal.

However, it should be noted that this is a limitation in [5] and does result in uncertainties when comparing single cellular data with data collected from trabeculae. Further to this, it does therefore cause uncertainty when comparing it to the results of this study.

One limitation of this study is the necessity to use data from multiple experimental studies to complete the biomarker characterisation for each atrial region. Due to differences in experimental protocol across different studies, it is unlikely the regional differences in the study accurately reflect true variability within and between regions. The RA region does clearly have increased variability compared with other regions in experimental data. This is no doubt a result of significantly more studies on the RA region than the others. It was decided to include this variability due to the fact that different experimental protocols will reduce the variability in cells in different ways. So, it is likely that this is more reflective of the degree of variability in the tissue than the variability observed in other regions with fewer studies. This suggests the other atrial regions are artificially reduced compared with the RA. This, however, does not impact the overall conclusions in this study. Inter-regional differences are likely to be impacted by the regional differences in variability as a result of inter-subject differences. This is mitigated by the reduction in regional differences in standard deviation for the APA and RMP biomarkers from experimental values to population values. For example, the standard deviation for the RMP in the regional populations varies between 1.5 and 2.4 mV, compared with a range of 1.4 to 12 mV in the experimental data. The variability in APD20, APD50, and APD90 across atrial regions remains large in the regional populations. It should be noted that this could artificially impact the inter-regional differences in results, but the observed overall impact across regions was consistent despite differences in population variability. Experimental observations in the action potential biomarkers for isolated cells showed differences in RMP [21], APD90 [5, 21], and APD50 [5]. This study showed that due to electrotonic coupling, the mean APD90 remained mostly unchanged compared with the single cellular models. A slight increase was observed in the mean APD90 for some regions, including the RAA. This is consistent with [5], whereby experimental observations of APD90 in AF remodelled trabeculae were 152 ms, compared with 149 ms in the isolated cells. In [21], there is a marked reduction in APD90 in the LA region in trabeculae compared with the single cellular data. In the RA trabeculae, the APD90 is in fact increased by 3 ms. This could be impacted by small sample sizes in the study but could also show differences in the impact of electrotonic coupling from region to region. Variability in APD90 occurs across the same region in the whole atrial model but remains consistent across 10 atrial models. This suggests the cellular heterogeneity, combined with heterogeneity in fibre direction and anisotropy across the region, causes further variability in repolarization across the atrial regions.

An interesting result from the simulations is the increase in the average value observed for the APD20 and APD50 biomarkers in the variable models because of the electrotonic coupling. Electrotonic coupling basically results in the prolongation of APD20 and APD50 because the electrotonic coupling is trying to find a new equilibrium to compensate for the heterogeneity. This is not observed in the homogeneous models because neighbouring cells in the same region are identical and therefore have the same steady state conditions and the same reaction to ion channel concentration. This goes to show that heterogeneity could result in a ‘new normal’ behaviour or new equilibrium in tissue or whole atrial behaviour that would not necessarily be observed in extracted cellular samples and single cellular samples.

The increase in APD50 causes a general reduction in AP triangulation at the tissue level with respect to that in isolation. This reduction in triangulation suggests that the electrotonic coupling, in the absence of gross conduction impairment, works as an antiarrhythmic mechanism. It is worth mentioning that this reduction in triangulation was not observed in the homogeneous atrial model. This may also indicate that inducing reentries in models with electrophysiological variability may be more difficult, in addition to presenting a smaller vulnerable window. For this reason, efforts are currently being placed on studying the effect that electrophysiological variability has on both reentry initiation and sustainability in homogeneous and heterogenous electrophysiological atrial models. Another interesting aspect of the simulations relates to the impact of tissue anisotropy in the presence of cellular heterogeneity, in particular in a scenario of such a complex fibre organisation as the atria. A careful study of this interaction would provide insight into the contribution of tissue anisotropy to changes in cellular behaviour, more precisely on the modulation of AP morphology, compared with the case of isotropic electrotonic coupling. Quantifying the impact of tissue anisotropy on the electrophysiological behaviour of individual cells and whole tissue/organ samples remains to be undertaken. This is particularly important in looking at the AF remodelled atria because intrinsic differences in APD and tissue anisotropy are increased in AF remodelling [22] and predispose the atria to rhythm disorders such as atrial fibrillation [25].

As mentioned previously, the reduction in triangulation due to variability would suggest that initiating a re-entry would be more difficult in the heterogeneous atria. Triangulation in the LA region was reduced from 89 to 69 ms as a result of heterogeneity, suggesting that the heterogeneity in this region would considerably reduce the ability to create a reentry. The AVR is the only region in which triangulation in the heterogeneous model is increased compared with the homogeneous model. No study to date has reported the impact of tissue heterogeneity on triangulation. Experimental data shows an increase in triangulation as a result of AF remodelling with respect to the healthy atria in single cellular observation [2], but no investigation into the impact of electrotonic coupling on triangulation has been reported. Both [2] and [14] report the variability observed in experimental data does not fully represent the properties and behaviour of cells in the whole atria. This is in part due to limitations of the cell isolation procedure for patch clamp experiments influencing experimental results. Additionally, patch clamp experiments do not include the impact of complex geometry, heterogeneity, and anisotropy observed in the atrial tissue. This study confirms that the combined impact of cellular heterogeneity, electrotonic coupling, and tissue anisotropy significantly changes the action potential morphology and variability in the tissue.

## Conclusion

Atrial activation, or the depolarization phase is not significantly impacted by the introduction of cellular heterogeneity in the atrial model. With only slight differences in activation across the atria and total activation times differing by 0–1 ms due to variability, it is clear that the depolarization across the atria is not impacted by variability when regional models are calibrated using isolated tissue samples. Observable and significant differences, however, were observed in the repolarization phase as a result of the introduction of cellular heterogeneity. Through the use of multiple heterogeneous atrial models, it has been shown that the impact of heterogeneity is consistent across multiple models and is not unique to a single heterogeneous model.

Through comparison of the original population of models used to create the heterogeneous atria and the tissue properties within the associated regions in the atria, the impact of electrotonic coupling was established. In all regions, electrotonic coupling resulted in a significant reduction in variability. Though the variability was significantly reduced, variability was still present. In regions such as the crista terminalis and Bachmann’s bundle, variability was less reduced because of electrotonic coupling and the RMP was significantly changed compared with the original population characteristics. This impact on smaller atrial regions, surrounded by tissues with different characteristics, showed that the impact of neighbouring tissues extends beyond the immediate boundary. Further to this, it can create a change in action potential morphology of tissues of smaller regions or in close proximity to tissue boundaries.

Overall, this study shows that electrotonic coupling does significantly impact variability, as expected, but does not completely eradicate it. This, combined with differences in repolarization as a result of the heterogeneity, suggest it is important to include cellular heterogeneity in atrial simulations. Furthermore, electrotonic coupling impacts all regions by reducing the variability in most biomarkers, but to varying degrees, with smaller regions maintaining more variability due to boundaries with other regions. Regional boundaries not only impact the variability but also significantly impact the mean value of all biomarkers in at least one region, therefore changing the characteristics of regions.

It has been shown that heterogeneity impacts the repolarization phase significantly. This could result in changes to the window of vulnerability across the atria and impact the propagation and maintenance of reentries. With APD determining the refractory period and the window of vulnerability in atrial tissue, it is a key determinant of the susceptibility to reentries. The observed changes in the repolarization across the atria as a result of cellular heterogeneity could significantly impact the progression and maintenance of reentries and atrial fibrillation. Further investigation into this could further improve the current understanding of the mechanisms of atrial fibrillation and lead to improved and more successful prevention and treatment methods.

Two main questions remain unanswered. Firstly, how cellular heterogeneity in the atria impacts the initiation, maintenance, progression, and characteristics of reentries in the AF remodelled atria. This question is particularly important in furthering the understanding of the mechanisms of atrial fibrillation and could potentially improve treatment methods. Secondly, determining the impact of anisotropy on the modulation of AP morphology in the presence of electrophysiological variability. This question could quantify the contributions of the electrotonic coupling and tissue anisotropy to the changes in biomarkers and tissue behaviour across the atria.

## Supplementary Information

Below is the link to the electronic supplementary material.

Supplementary file1 (DOCX 177 KB)

## Abbreviations

AF: Atrial fibrillation
APA: Action potential amplitude
APD: Action potential duration
APD20: Action potential duration at 20% repolarization
APD50: Action potential duration at 50% repolarization
APD90: Action potential duration at 90% repolarization
AVR: Atrio-ventricular rings
BB: Bachmann bundle
BCL: Basic cycle length
CS: Coronary sinus
CT: Crista terminalis
CV: Conduction velocity
FO: Fossa ovalis
ISTMO: Isthmus
LA: Left atria
LAA: Left atrial appendage
PM: Pectinate muscles
PV: Pulmonary veins
RA: Right atria
RAA: Right atrial appendage
RMP: Resting membrane potential
POM: Population of models
SAN: Sinoatrial node
TRI: Triangulation

## References

[1] Workman AJ, Kane KA, Rankin AC (2008). Cellular bases for human atrial fibrillation. Heart Rhythm.

[2] Bosch RF, Nattel S (2002). Cellular electrophysiology of atrial fibrillation. Cardiovasc Res.

[3] Nattel S (2003). Atrial electrophysiology and mechanisms of atrial fibrillation. J Cardiovasc Pharmacol Ther.

[4] Schotten U, Verheule S, Kirchhof P, Goette A (2020) Pathophysiological mechanisms of atrial fibrillation: a translational appraisal. 265–325. 10.1152/physrev.00031.2009 10.1152/physrev.00031.2009 21248168

[5] Dobrev D, Graf E, Wettwer E, Himmel HM, Hála O, Doerfel C (2001). Molecular basis of downregulation of G-protein – coupled. Circulation.

[6] Gaspo R, Bosch RF, Bou-Abboud E, Nattel S (1997). Tachycardia-induced changes in Na+ current in a chronic dog model of atrial fibrillation. Circ Res.

[7] Loose S, Mueller J, Wettwer E, Knaut M, Ford J, Milnes J (2014). Effects of IKur blocker MK-0448 on human right atrial action potentials from patients in sinus rhythm and in permanent atrial fibrillation. Front Pharmacol.

[8] Ferrer A, Sebastián R, Sánchez-Quintana D, Rodríguez JF, Godoy EJ, Martínez L (2015). Detailed anatomical and electrophysiological models of human atria and torso for the simulation of atrial activation. Panfilov A V, editor. PLOS ONE.

[9] Tixier E, Lombardi D, Rodriguez B, Gerbeau JF (2017) Modelling variability in cardiac electrophysiology: a moment-matching approach. J R Soc Interface 14.10.1098/rsif.2017.0238 10.1098/rsif.2017.0238 PMC5582121 28835541

[10] Aronis KN, Ali RL, Liang JA, Zhou S, Trayanova NA (2019) Understanding AF mechanisms through computational modelling and simulations. Arrhythm Electrophysiol Rev, Radcliffe Cardiology 8:210–9. 10.15420/aer.2019.28.2 10.15420/aer.2019.28.2 PMC6702471 31463059

[11] Heijman J, Voigt N, Nattel S, Dobrev D (2014). Cellular and molecular electrophysiology of atrial fibrillation initiation, maintenance, and progression. Circ Res.

[12] Heidenreich EA, Ferrero JM, Doblaré M, Rodríguez JF (2010). Adaptive macro finite elements for the numerical solution of monodomain equations in cardiac electrophysiology. Ann Biomed Eng.

[13] Iwasaki YK, Nishida K, Kato T, Nattel S (2011). Atrial fibrillation pathophysiology: implications for management. Circulation.

[14] Adeniran I, Maciver DH, Garratt CJ, Ye J, Hancox JC, Zhang H (2015). Effects of persistent atrial fibrillation- induced electrical remodeling on atrial electro-mechanics - insights from a 3D model of the human atria. PLoS ONE.

[15] Kim BS, Kim YH, Hwang GS, Pak HN, Lee SC, Shim WJ (2002). Action potential duration restitution kinetics in human atrial fibrillation. J Am Coll Cardiol, Elsevier Masson SAS.

[16] Martinez-Mateu L, Romero L, Ferrer-Albero A, Sebastian R, Rodríguez Matas JF, Jalife J (2018). Factors affecting basket catheter detection of real and phantom rotors in the atria: a computational study. PLoS Comput Biol.

[17] Sánchez C, Bueno-Orovio A, Pueyo E, Rodríguez B (2017). Atrial fibrillation dynamics and ionic block effects in six heterogeneous human 3D virtual atria with distinct repolarization dynamics. Front Bioeng Biotechnol.

[18] Tobón C, Ruíz C, Rodríguez JF, Hornero F, Ferrero JM, Saiz J (2010) Vulnerability for reentry in a three dimensional model of human atria: a simulation study. 2010 Annual International Conference of the IEEE Engineering in Medicine and Biology Society, EMBC’10, 224–7. 10.1109/IEMBS.2010.5627810 10.1109/IEMBS.2010.5627810 21096955

[19] Tobón C, Ruiz-villa CA, Heidenreich E, Romero L, Hornero F (2013) A three-dimensional human atrial model with fiber orientation. Electrograms and arrhythmic activation patterns relationship. 8. 10.1371/journal.pone.0050883 10.1371/journal.pone.0050883 PMC3569461 23408928

[20] Anyukhovsky EP, Sosunov EA, Chandra P, Rosen TS, Boyden PA, Danilo P (2005). Age-associated changes in electrophysiologic remodeling: a potential contributor to initiation of atrial fibrillation. Cardiovasc Res.

[21] Li D, Zhang L, Kneller J, Nattel S (2001). Potential ionic mechanism for repolarization differences between canine right and left atrium. Circ Res.

[22] Sánchez C, Britton OJ, Muszkiewicz A, Gemmell P, Rodriguez B, Passini E (2015). Variability in cardiac electrophysiology: using experimentally-calibrated populations of models to move beyond the single virtual physiological human paradigm. Prog Biophys Mol Biol.

[23] Osaka T, Itoh A, Kodama I (2000). Action potential remodeling in the human right atrium with chronic lone atrial fibrillation. PACE - Pacing Clin Electrophysiol.

[24] Ridler ME, Lee M, McQueen D, Peskin C, Vigmond E (2011) Arrhythmogenic consequences of action potential duration gradients in the atria. Can J Cardiol, Elsevier Inc. 27:112–9. 10.1016/j.cjca.2010.12.002 10.1016/j.cjca.2010.12.002 21329870

[25] Trayanova NA (2014). Mathematical approaches to understanding and imaging atrial fibrillation: Significance for mechanisms and management. Circ Res.

[26] Zrenner B, Ndrepepa G, Karch MR, Schneider MAE, Schreieck J, Schömig A (2001). Electrophysiologic characteristics of paroxysmal and chronic atrial fibrillation in human right atrium. J Am Coll Cardiol.

[27] Ravens U, Fernandez-Aviles F, Rodrigo M, Liberos A, Guillem MS, Rodriguez B (2016). Balance between sodium and calcium currents underlying chronic atrial fibrillation termination: an in silico intersubject variability study. Heart Rhythm, Elsevier.

[28] Burrage P, Lawson BAJ, Drovandi CC, Burrage K, Pettitt AN, Cusimano N (2016). Sampling methods for exploring between-subject variability in cardiac electrophysiology experiments. J R Soc Interface.

[29] Elliott J, Belen MK, Mainardi L, Rodriguez Matas JF (2021) A comparison of regional classification strategies implemented for the population based approach to modelling atrial fibrillation. Mathematics 9. 10.3390/math9141686

[30] Lawson BAJ, Drovandi CC, Cusimano N, Burrage P, Rodriguez B, Burrage K (2018) Unlocking data sets by calibrating populations of models to data density: a study in atrial electrophysiology. Sci Adv 4. 10.1126/sciadv.1701676 10.1126/sciadv.1701676 PMC5770172 29349296

[31] Liu X, Muszkiewicz A, Rodriguez B, Casadei B, Lawson BAJ, Bueno-Orovio A (2017). From ionic to cellular variability in human atrial myocytes: an integrative computational and experimental study. Am J Physiol-Heart Circ Physiol.

[32] Courtemanche M, Ramirez RJ, Nattel S (1998). Ionic mechanisms underlying human atrial action potential properties: insights from a mathematical model. Am J Physiol.

[33] Bosch RF, Zeng X, Grammer JB, Popovic K, Mewis C (1999). Ionic mechanisms of electrical remodeling in human atrial fibrillation. Cardiovascular Research.

[34] Burashnikov A, Mannava S, Antzelevitch C (2004). Transmembrane action potential heterogeneity in the canine isolated arterially perfused right atrium: effect of IKr and I Kur/Ito block. Am J Physiol - Heart Circ Physiol.

[35] Feng J, Yue L, Wang Z, Nattel S (1998). Ionic mechanisms of regional action potential heterogeneity in the canine right atrium. Circ Res.

[36] Hara M, Shvilkin A, Rosen MR, Danilo P, Boyden PA (1999). Steady-state and nonsteady-state action potentials in fibrillating canine atrium: abnormal rate adaptation and its possible mechanisms. Cardiovasc Res.

[37] Wang ZG, Pelletier LC, MT, SN (2002) Effects of a novel class III antiarrhythmic agent. 66:185–91. 10.1161/01.CIR.82.1.274

[38] Pau D, Workman AJ, Kane KA, Rankin AC (2007) Electrophysiological and arrhythmogenic effects of 5-hydroxytryptamine on human atrial cells are reduced in atrial fibrillation. J Mol Cell Cardiol, Elsevier Inc. 42:54–62. 10.1016/j.yjmcc.2006.08.007 10.1016/j.yjmcc.2006.08.007 PMC2526346 16989857

[39] Sánchez C, Bueno-Orovio A, Wettwer E, Loose S, Simon J, Ravens U et al (2014) Inter-subject variability in human atrial action potential in sinus rhythm versus chronic atrial fibrillation. PLoS ONE 9. 10.1371/journal.pone.0105897 10.1371/journal.pone.0105897 PMC4144914 25157495

[40] Yue L, Feng J, Gaspo R, Li GR, Wang Z, Nattel S (1997). Ionic remodeling underlying action potential changes in a canine model of atrial fibrillation. Circ Res.

[41] Workman AJ, Kane KA, Rankin AC (2001). The contribution of ionic currents to changes in refractoriness of human atrial myocytes associated with chronic atrial fibrillation. Cardiovasc Res.

[42] Cha TJ, Ehrlich JR, Zhang L, Chartier D, Leung TK, Nattel S (2005). Atrial tachycardia remodeling of pulmonary vein cardiomyocytes: comparison with left atrium and potential relation to arrhythmogenesis. Circulation.

[43] Aslanidi OV, Colman MA, Stott J, Dobrzynski H, Boyett MR, Holden AV (2011). 3D virtual human atria: a computational platform for studying clinical atrial fibrillation. Prog Biophys Mol Biol, Elsevier Ltd.

[44] Krueger MW, Dorn A, Keller DUJ, Holmqvist F, Carlson J, Platonov PG (2013). In-silico modeling of atrial repolarization in normal and atrial fibrillation remodeled state. Med Biol Eng Compu.

[45] Romero L, Pueyo E, Fink M, Rodríguez B (2009). Impact of ionic current variability on human ventricular cellular electrophysiology. Am J Physiol - Heart Circ Physiol.

[46] Lemery R, Birnie D, Tang ASL, Green M, Gollob M, Hendry M (2007). Normal atrial activation and voltage during sinus rhythm in the human heart: an endocardial and epicardial mapping study in patients with a history of atrial fibrillation. J Cardiovasc Electrophysiol.

