# Supplementary material for: Cellular heterogeneity and repolarisation across the atria: an in silico study
Source: Med Biol Eng Comput. 2022 Sep 15;60(11):3153–68. doi: 10.1007/s11517-022-02640-x (PMC9537222; doi:10.1007/s11517-022-02640-x)
Supplement: Supplementary file 1 — Supplementary file1 (DOCX 177 KB) [file 11517_2022_2640_MOESM1_ESM.docx]

Supplementary Material: Cellular Heterogeneity and Repolarization Across the Atria: an in-Silico Study

Jordan Elliott^1*^, Luca Mainardi^2^, Jose Felix Rodriguez Matas^1*^

^1^ Department of Chemical and Material Engineering, Politecnico Di Milano, Milan, Italy, 20133;

^2^ Department of Electronic, Information and Bioengineering, Politecnico Di Milano, Milan, Italy, 20133;

***
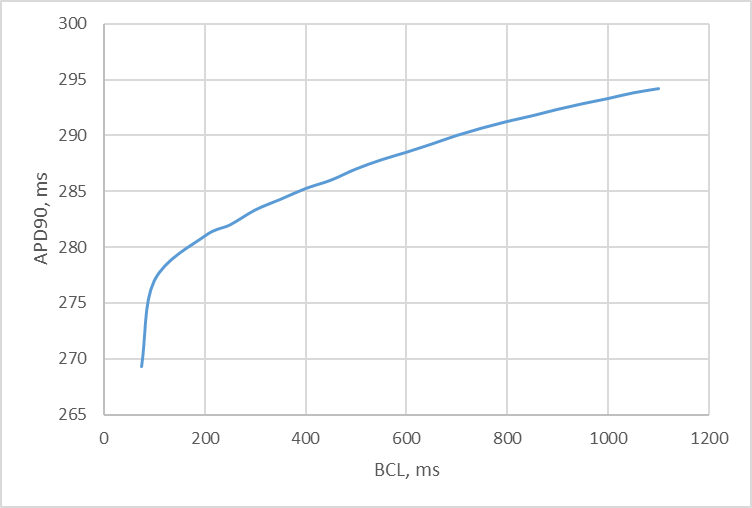
***

Figure S1 Restitution curve for the standard Courtemanche-Ramirez-Nattel single cellular model.

| ***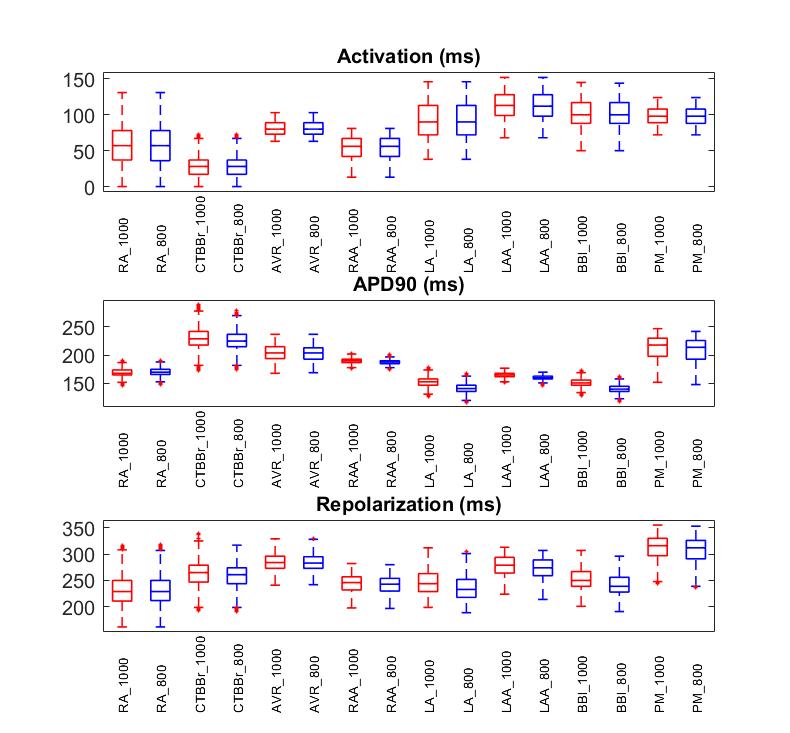***  (A) | ***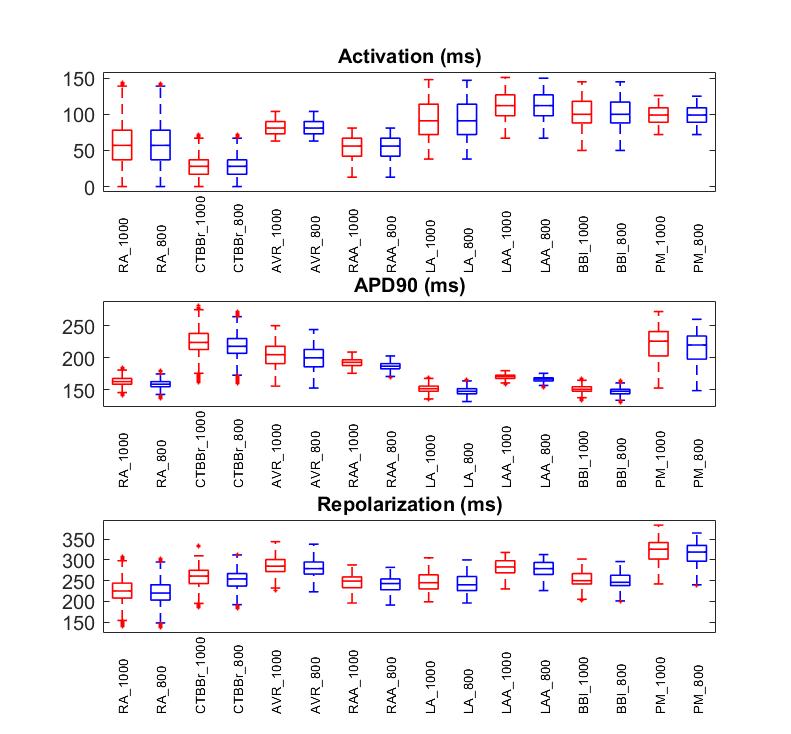***  (B) |
| --- | --- |

Fig S2. Effect of stimulation frequency on AP markers Results for CL=1000ms in red and for CL=800ms in blue. A) Homogeneous model; B) heterogeneous model.
